# Psychosocial and financial impacts for carers of those with eating disorders in New Zealand

**DOI:** 10.1186/s40337-022-00565-2

**Published:** 2022-03-15

**Authors:** Lois J. Surgenor, Shistata Dhakal, Roma Watterson, Brendan Lim, Martin Kennedy, Cynthia Bulik, Nicki Wilson, Karen Keelan, Rachel Lawson, Jennifer Jordan

**Affiliations:** 1 Department of Psychological Medicine, University of Otago, Christchurch, New Zealand; 2 School of Psychology, Speech and Hearing, University of Canterbury, Christchurch, New Zealand; 3 PeopleSense, Altius Group, Canberra, Australia; 4 Department of Pathology and Biomedical Science, University of Otago, Christchurch, New Zealand; 5 Department of Psychiatry, University of North Carolina at Chapel Hill, Chapel Hill, USA; 6 Department of Medical Epidemiology and Biostatistics, Karolinska Institutet, Stockholm, Sweden; 7 Department of Nutrition, University of North Carolina at Chapel Hill, Chapel Hill, USA; 8 Eating Disorders Association of New Zealand, Wellington, New Zealand; 9 Cancer Control Agency, Te Aho o Te Kahu, Christchurch, New Zealand; 10 South Island Eating Disorders Service, Canterbury District Health Board, Christchurch, New Zealand; 11 Specialist Mental Health Clinical Research Unit, Canterbury District Health Board, Christchurch, New Zealand

**Keywords:** Eating disorders, Carer burden, New Zealand

## Abstract

**Background:**

Eating disorders (ED) can have profound effects on family members and carers. These impacts can be experienced across multiple domains and may contribute to the maintenance of ED symptoms. In the absence of any New Zealand studies quantifying this, and given country-specific differences in access to care and treatment, this study explores the psychosocial and economic impacts on those caring for someone with an ED in New Zealand.

**Methods:**

Carers (N = 121) of those who had, or still had, a self-reported ED (82.6% anorexia nervosa) completed an online survey open between December 2016 and October 2020, adapted to the New Zealand context. Questions addressed ED recency and recovery status of the individual cared for, treatment access, and the financial and psychosocial impact on the carer. Data analysis included descriptive statistics, with financial cost data converted to the equivalent of 2020 New Zealand dollars.

**Results:**

Most (88.6%) recruited carers reported still caring for someone with ED symptoms of varying severity. A majority reported difficulty accessing treatment for the person they cared for, with a sizable minority (45%) paying for private treatment, despite few having private insurance. Carer losses typically included reduced income and productivity, travel costs, and other miscellaneous costs. Carers reported significant psychosocial impacts across a range of dimensions including family life, interpersonal relationships, and their own personal well-being.

**Conclusions:**

Carers in New Zealand report impacts which are far reaching and longstanding, covering their own personal and interpersonal well-being and that of those around them. While most of those they care for get access to public (free) treatment at some time or another, the wider financial and economic impacts on carers are significant, and likely to take years to recoup. Though not unique to EDs, interventions and supports for carers are much needed in New Zealand, alongside more comprehensive research methodology to further determine positive and other impacts of EDs over the long course of the caregiving role.

**Highlights:**

- A majority reported difficulty accessing treatment for the person they cared for
- 45% paid for private treatment, despite few having private insurance
- Carers reported reduced income and productivity, travel costs, and other costs.
- Carers reported significant psychosocial impacts on family life, interpersonal relationships, and their own personal well-being.
- Carers provide a pivotal role in supporting treatment and recovery in their family member with the
- These findings will be relevant for funders and service providers in developing further approaches to address barriers and gaps in service provision to reduce impacts on carers, and as a result, those with eating disorders.

## Plain English summary

Caring for a person with any significant health or disability condition can have a harmful effect on the carer’s own wellbeing. This study estimates this burden of caring for people with either ongoing ED symptoms or who have recovered, in New Zealand. The study recruited 121 carers, of whom almost all (97.5%) were parents, with most caregivers reporting impacts as this related to caring for someone with anorexia nervosa (82.6%).

Overall, the results found widespread impacts on carers, particularly impacts on multiple relationships in their life, especially family relationships. These impacts extended to reporting harmful effects on the relationship with the person with the ED. Carers are also burdened by difficulties accessing treatment and wider economic impacts such as loss of income and productivity at work. As New Zealand’s health system is relatively unique and this is the first quantitative study in New Zealand exploring ED carer impacts, larger studies are needed to capture the full extent of this in order to better meet these needs both at a health service and government level.

## Introduction

The terms “burden of illness” or “burden of care” are widely used in the health economics literature. These terms aim to quantify a broad range of impacts on those affected by the condition and those who care for them [1]. Commonly, research in these areas may identify unmet needs, and as a result, suggest resources or interventions that may alleviate these impacts [2]. In this paper we choose to refer in most part to “carer impacts” as a more neutral term.

The literature on carer impacts is a universal issue across many different illnesses and disabilities. Broadly defined, carer impacts can include any negative impact in carers caused by the demands of providing care to a person with an illness or disability. Thus, carer impacts can be expressed across multiple domains including direct and indirect financial impacts, time and resource burden and psychological, physical and relationship impacts. Commonly, research has clustered these domains into objective or subjective burden components [3]. The former typically refers to tangible and quantifiable impacts such as the hours spent carrying out carer tasks and other actual disruptions; subjective burden typically refers to perceived psychological and relational/lifestyle/family impacts. It has been suggested that the two components are only moderately associated with each other which supports the need to study both when assessing caregiver burden [4].

Other researchers have suggested that the relationship between these components is complex and dynamic [5]. For example, long hours spent carrying out carer tasks may disrupt other relationships. Ultimately both components may have an impact on health domains including physical health. Positive aspects of being a caregiver (‘carer rewards’) have also been acknowledged as important [6] though this area is less frequently included in carer burden studies.

Further, carer impacts have been associated with gender, generally finding that female caregivers, across a range of health conditions including eating disorders, may experience greater impacts [7, 8]. Different theories have been put forward to explain possible differences in caregiver impacts including those relating to gender role expectations of society [9]. However, meta-analyses have produced mixed findings [10] about additional impacts for women, suggesting that there are nuanced factors still to be explored.

While it can be assumed that longer duration and more severe disorders are associated with higher burden of care, carer impacts also may have an impact on the care provided to the person being cared for. For example, across a range of health conditions, the consequences of the impact on carers may contribute to poorer home care e.g. in the context of palliative care [11] or to the maintenance of symptoms or even interfere with treatment. This has been discussed in relation to EDs, especially in the area of AN [12].

### Costs of carer impacts in eating disorders

Several large studies have reported the broad social and financial/economic costs of eating disorders, though different approaches have been taken to quantify costs. In Australia, the ‘Butterfly Report’ [13] found that carers (n = 525) spent on average 12.4 h per week in caring for a person with an eating disorder, which translated to an estimated $8.54 million opportunity cost in lost wages at that time. No further breakdown of carer impacts was provided.

In the United Kingdom (UK) [14], carers (n = 82) were estimated to incur an average financial burden of £2800 per year, and another £5950 per annum in relation to other economic costs such as time off work and education costs. In terms of subjective burden, the vast majority of carers reported significant or very significant impacts on family and social life, overall well-being and quality of life, though these data reported on caregivers for a range of eating disorders.

Two more recent studies have been published on the economic burden. In Taiwan [15], people with eating disorders had significantly higher health service utilisation and costs when compared with non-eating disorder controls, noting that people with AN had the highest utilisation and costs of those with eating disorders. Carer burden was not included in that study, though it was recommended to do so in future research. Given the frequent age of onset in adolescence, much of this cost is likely to be borne by caregivers. While focusing on focusing on patient and treatment costs, a study in the United States [16] included the opportunity cost of caregiver’s time, estimating this at $22.50 USD per hour.

In summary, the overall literature on burden of disease for eating disorders is significant, and is possibly increasing disproportionately to that of other psychiatric disorders [17]. The large economic burden impact studies have not routinely included carer impacts and very few studies have explored positive aspects of the carer role, which in the context of eating disorders has included improved family alliance and increased empathy [18, 19]. A larger body of research has more directly focused on the mental health and general wellbeing of carers, and in regard to AN, these are discussed below.

### Impacts on carers of those with anorexia nervosa

AN is an extremely serious disorder with high rates of comorbidity [20] along with all-cause and suicide mortality [21, 22]. AN has a protracted illness course for many [23], with estimates of around a third relapsing after treatment [24]. Long-term studies suggest only a third have recovered after 10 years [25], though this may increase to two-thirds after 30 years [26]. All these findings suggest that carer impacts for this particular eating disorder are potentially long-term. The early age of onset compared with other eating disorders contributes to this, as does illness duration and symptom severity [27, 28] though this is not a universal finding [29]. Around a third of parent carers of adolescents with AN exhibit moderate to severe levels of distress [8], with mothers being especially impacted. This disproportional maternal burden has been found in other studies [30]. When compared against other psychiatric disorders, AN extracts an especially high negative impact on carers [31].

There are additional reasons for documenting, understanding and addressing the impact on AN carers. While the relationship between family disturbance and AN is still far from clear [32], there is some consensus that vicious cycles can develop such that secondary strained family relationship dynamics may evolve over time. For example, overly anxious caregivers may find themselves taking more control of mealtimes, thereby leading to battles for control and escalated tensions because of this. Further, carer burden in AN can pose a risk for accommodating (adapting to) AN and thereby inadvertently maintaining the condition [19, 31]. For example, carers who become overwhelmed, or blame themselves, may find themselves coping by modifying routines in the household in order to assist food avoidance or exercise rituals. In response to this, care-giver issues have been more explicitly included in the new Maudsley model [33]. Subsequent research has explored the impact of providing direct interventions to reduce this carer burden [34–36].

Despite the volume of research increasing, the area of carer burden in eating disorders remains under investigated [2] and there are no New Zealand quantitative studies to the best of our knowledge. International studies shed some light on the likely impacts on carers in New Zealand, though differing health, cultural and social support/funding contexts will limit generalisations. Even in closely aligned countries, access routes to private (and public) mental health services can differ significantly. For example, in Australia the universal public insurance programme [37] facilitates access to private services, whereas in New Zealand access to private services is either self-funded or through private insurance. Further, if private insurance is accessible, typically this may have a cap per year (e.g., one of New Zealand’s largest private insurance companies = maximum of $600 per year for clinical psychologist [38] which would fund only a few appointments.

Distress due to the inability to access care in a timely manner is a major theme in the accounts of carers of those with AN [2, 39]. Though these are not New Zealand studies, submissions to the New Zealand Government Mental Health Inquiry highlight treatment inaccessibility as a core issue [40].

Another difference in New Zealand compared with some countries is that a single government agency (“Pharmac”) decides which medicines are funded. This means that costs of some medications, which may be prescribed for people with eating disorders, may be subsidised.

This study is part of a larger project, the Costs of Eating Disorders in New Zealand, examining both consumer and carer perspectives [41], running alongside a qualitative study [18].

## Method

### Measurement of carer burden

Carer impacts in eating disorders has been measured in multiple ways including standardised self-report inventories specifically developed for eating disorder (e.g., Eating Disorders symptom impact scale (EDSIS) [42] and more general scales (Involvement Evaluation Questionnaire-European Version (IEQ-EU [43], as well as single questions to probe the impact severity (e.g., “very little impact” to “very significant impact) across different life domains [14]. With permission, the current study used and adapted questions from the latter, using the REDCap consortium platform [44] and based on the surveys conducted by national eating disorder support organisations in the UK [45] and Australia [13].

In summary, the questions assessing psychosocial impacts were those used in the original survey [14]. The question was “What degree of impact has caring for our family member had on each of these areas of your own life?” rated on a 5-point Likert scale from Very significant impacts to Very little or no impact, with a not applicable option. The items they rated were: Your social life; Your overall wellbeing and quality of life; Your participation and productivity at work (if you are in work); Your engagement and attainment in your education. We also added the following items to the original list: Your family life in general; Your relationship with your family member with the eating disorder; Your relationship with any other children you have; Your relationship with partner/ spouse; Other family relationships. The main adaptions related to the New Zealand health provision and demographic context.

A copy of the survey is available upon request. This current paper uses data reported by carers in relation to their affected individual with the eating disorder.

Carer-specific data included demographic information, their relationship to the affected individual (AI) with an eating disorder, and psychosocial impacts of the eating disorder on carers’ relationships and quality of life. Financial impacts for carers included estimated lost income, reduced hours of work and productivity, direct costs of treatment over and above any public funding, whether they had health insurance to fund treatment, needing to take out loans for treatment costs, and costs such as transport, accommodation and relocation expenses. Median yearly income data and costs of living adjustments for each year were obtained from Statistics New Zealand data sites [46]

Information about affected individuals included their demographic characteristics, ED diagnoses and aspects of treatment history, as reported by the carer participants.

### Recruitment

Data collection occurred from December 2016 to October 2020. Recruitment methods were repeated over the course of the study. These included postings about the study on the University of Otago, Christchurch Eating Disorder Research Facebook page, community noticeboards and other eating disorder related Facebook pages, and student group Facebook pages, as well as the University of Otago Twitter feed. Wider publicity occurred through the Eating Disorders Association of New Zealand (EDANZ) webpage and their mailing list. Hard copy posters with a link to our website were distributed across New Zealand in a broad range of community locations. The anonymous online survey was delivered via REDCap [44].

### Participants

#### Inclusion criteria

Participants were New Zealand residents aged 16 years or over of any gender or ethnicity who had cared for someone with a self-reported clinically significant ED. Brief definitions were provided using key DSM-5 criteria. For example, for AN, the following information was provided: “Food restriction leading to significantly low body weight (according to age, sex, development trajectory, and physical health); AND an intense fear of gaining weight or becoming fat, or persistent behaviour that interferes with weight gain, even when underweight; AND a disturbed body image experience, undue influence of weight and shape on self-evaluation, or not recognising the seriousness of low bodyweight.”

Participants had to be able to read and converse in English and to consent to participate. Participants could select yes, no or probable for the following DSM-5 diagnoses or symptoms for their affected individual (AI): anorexia nervosa; bulimia nervosa (BN); binge eating disorder (BED); OSFED diagnoses (atypical anorexia nervosa, atypical bulimia nervosa with low frequency and/ or limited duration; atypical binge eating disorder with low frequency and/ or limited duration; purging disorder; night eating syndrome). Treatment for the AI had to have occurred within New Zealand. Where an AI had received treatment in NZ and also overseas, only NZ data were included. The only exclusion criteria were that the carer was under the age of 16 years, or living outside New Zealand.

### Ethics

Prospective participants read the online information sheet online and consented by ticking a box to continue to the survey. The study had ethical approval from the relevant approving agency (Northern B Health and Disability Ethics Committee, New Zealand 16/NTB/189/AM01).

### Recoding diagnoses

Participants could endorse multiple diagnoses, but were asked to indicate which was the most significant ED for them. If they did not nominate the most significant, we imputed that data point using the following criteria: a threshold diagnosis was selected over a subthreshold OSFED diagnosis, and priority between threshold diagnoses was in this order: AN as first priority, then BN then BED.

### Statistics

Data were analysed using SPSSv27. Missing or contradictory information was checked by reading through the entire completed survey completed by the carer including free text entries and related variables. If there was insufficient information to impute, the field was left as missing. Free text data entries from the surveys were converted to a common metric (for example, to weeks). Costs reported from previous years were converted from the relevant year in which treatment occurred to 2020 New Zealand dollars using an online conversion calculator [47]

Only descriptive information (percentages, median, ranges) are reported for the sample as a whole. Medians were reported rather than means where there were skewed demographic and economic data. The predominance of an AN diagnosis in the AI (82.6% of the sample) precluded diagnostic between-group analyses.

## Results

### Characteristics of the carer sample and their affected individuals

Included in the study were n = 121 carers of an affected individual (AI) with an eating disorder.

### Carer demographic information

Carer participants were predominantly female (92.6%), and of European ethnicity (NZ born European 89.3%), Māori (3.5%) and Other (7.2%). The median age was 51 (range 17–73) years. Almost all were parents (97.5%) but two were spouses/partners (1.7%) and one (0.8%) was the adult child of a parent with an ED. At the time of completing the survey, 37.2% were working full-time, 32.2% were working part-time, and 14% were self-employed. Other occupations were stay at home parent (5.8%), unpaid carer (4.1%), student or retired (2.5% respectively). None were unemployed.

Table 1 presents the descriptive data about the AIs for whom the carer participants were reporting.

**Table 1:** Characteristics of affected individuals, as reported by their carer participants (N = 121 unless otherwise stated)

Affected individuals	n (%) or median (range)
*Age*	18 years (12–51)
*Gender*	
Female	101 (83.5%)
Male	9 (7.4%)
Other/Prefer not to say	11 (9.1%)
*Diagnosis*	
Anorexia nervosa	100 (82.6%)
Bulimia nervosa	10 (8.3%)
Binge eating disorder	1 (.8%)
Other specified feeding and eating disorders	10 (8.3)%
*Eating disorder history*	
Age of AI when carer noticed ED (n = 120)	15 years (7–35 years)
Duration of ED (includes current)	2.5 years (0.5 to 31 years)
*Recency of eating disorder (ED)*	
Within the past 5 years	114 (94.2%)
5–10 years ago	5 (4.1%)
Greater than 10 years	2 (1.7%)
*Eating disorders status*	
Still have ED	37 (30.6%)
Significant ED but somewhat improved	26 (21.5%)
Some symptoms but much improved	44 (36.4%)
Recovered	14 (11.6%)
Subsequent treatment episode for ED	44 (43.1%)
*Treatment*	
Difficulty finding/accessing treatment (n = 113)	
Not difficult	12 (9.9%)
Easier than other conditions	6 (5%)
Same as other conditions	19 (15.7%)
Difficult	32 (26.4%)
Very difficult	44 (36.4%)
Treatment received (multiple options could be endorsed)	
No treatment (n = 118)	3 (3.4%)
Public funded treatment only (n = 121)	88 (72.7%)
Privately-funded treatment only (n = 121)	11 (9%)
Both public and private funded treatment (n = 121)	14 (11.6%)
Treatment type	
Inpatient	55 (45.5%)
Day-patient	13 (10.7%)
Outpatient	69 (57%)

### Affected Individual (AI) characteristics

For the whole sample, the median age of AIs was 18 years (range 12–51 years), and the majority were female (83.5%) with 7.4% males and 9.1% endorsing other gender or preferring not to say. Ethnicity data were not collected about the AIs. Regarding diagnoses, as noted above, most of the AIs reported on had a diagnosis of AN (82.6%, n = 100), with other diagnoses far less prevalent in the sample: BN (8.3%, n = 10), BED (0.8%, n = 1) and OSFED (10%, n = 8).

The median age of the AI when the carer noticed the ED was 15 years (range 7–35 years). Almost all (94.2%) reported on an ED that had occurred or was still present within the past 5 years. The median duration (including those who were symptomatic at the time of the survey) was 2.5 years. Only 11.6% had recovered at the time of the survey, meaning most were still symptomatic to some extent. The “current symptoms” categories included: some symptoms but much improved (36.4%); significant ED but much improved (21.5%); and still have a current ED diagnosis (30.6%). Reported recurrence of the ED occurred in 43.1% of the whole sample.

### Treatment

There were 113 responses to the treatment questions, so percentages are of those responding. Three AIs reported on by the carer sample had never received treatment (AN n = 1, OSFED n = 2). However, two thirds (67.3%) of the carers reported that it was difficult or very difficult to get help for the AI’s eating disorder. Of those answering these questions, 72.7% of the AIs had received public-funded treatment, and a further 11.6%) received both public and privately-funded treatment. Eleven people (9%) had only privately funded treatment. Within the sample, 45.5% (n = 55) reported that their AIs had received inpatient treatment, 57% (n = 69) received outpatient treatment while only 10.7% (n = 13) received day-patient treatment.

### Psychosocial impacts of the ED for carers

**Fig. 1:**
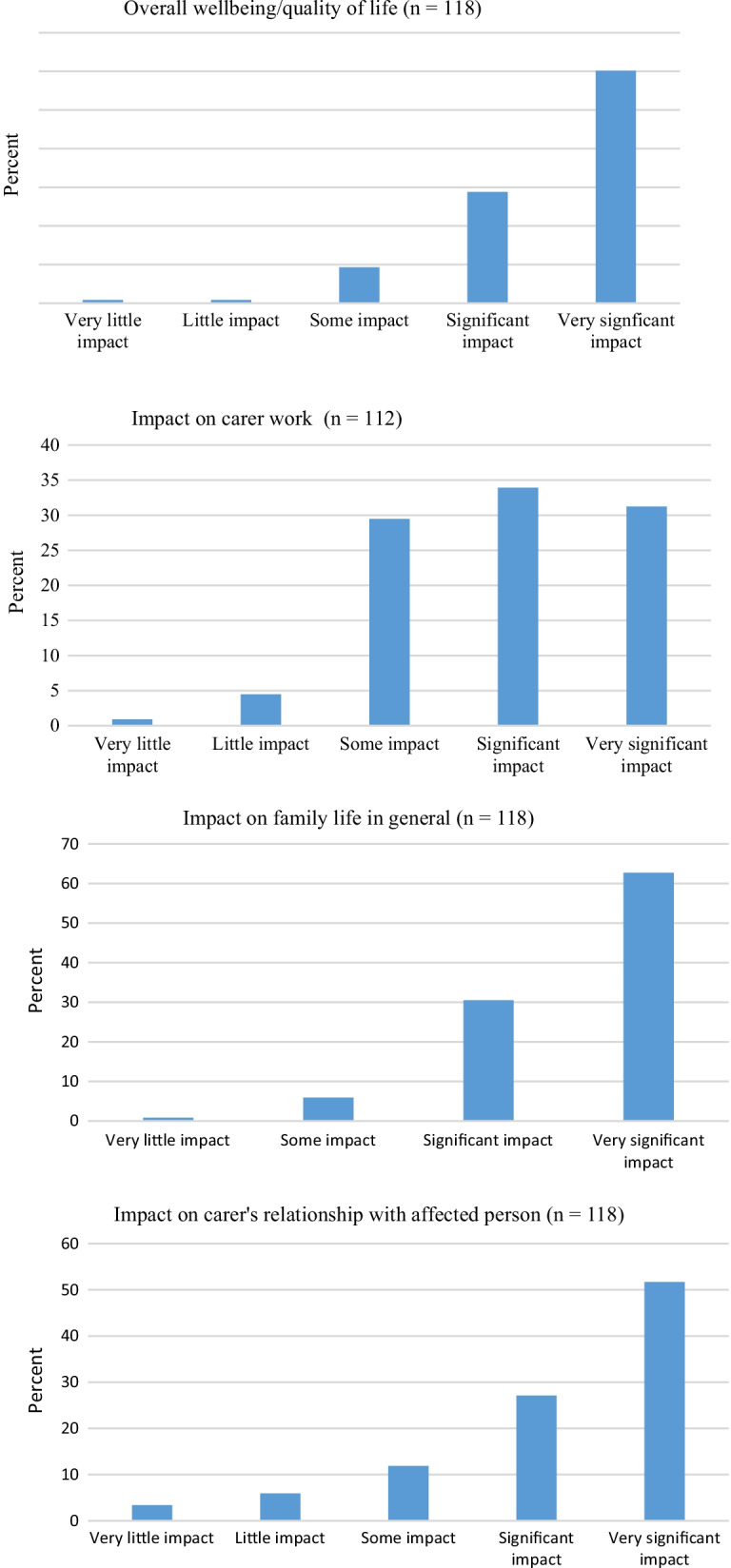

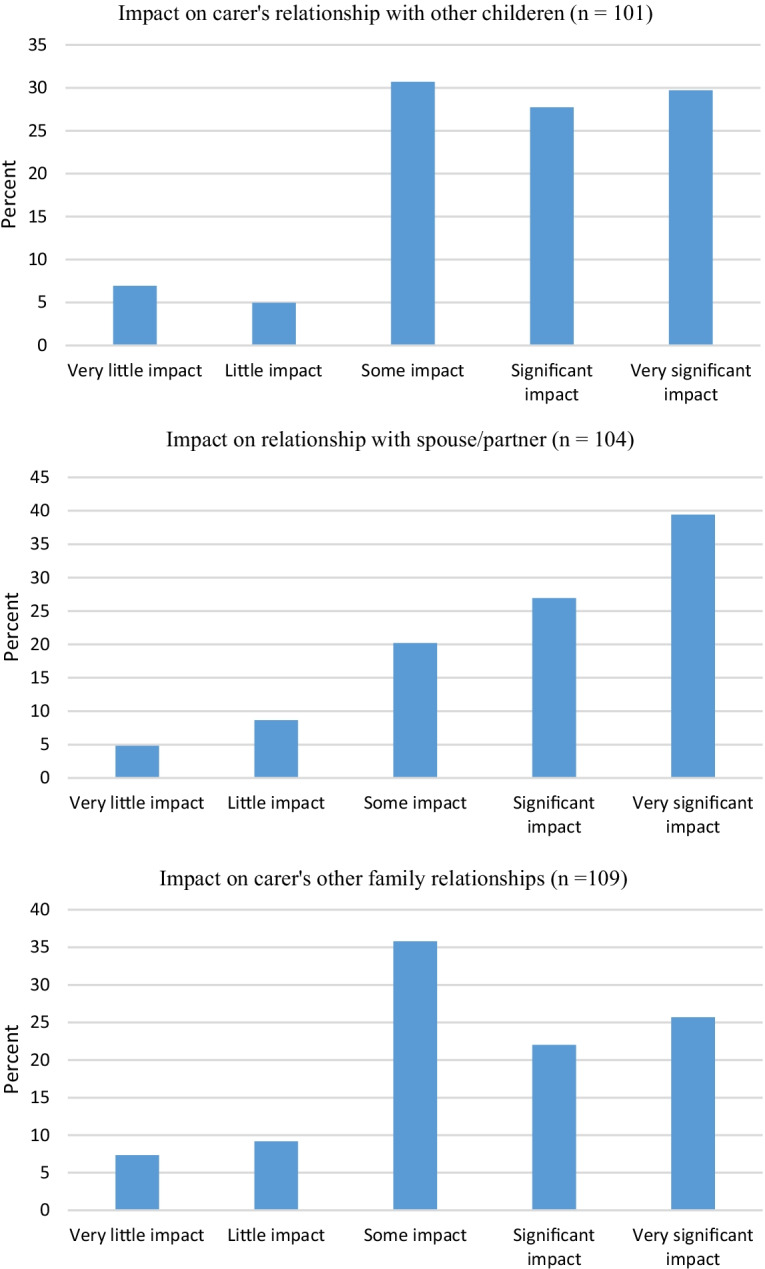
Psychosocial impacts on carers

As a group, carers reported significant psychosocial impacts arising from the ED (Fig. 1).

In this respect, over 90% (93.2%) of carers reported “significant” or “very significant” impact on family life generally, with over 80% (84.2%) reporting “significant” or “very significant” impacts on social/family life and, to a lesser extent, broader family relationships were still significantly or very significantly impacted by almost half (47.7%). Significant or very significant impacts on relationships with their spouse/partner occurred in two-thirds (66.3%) of carers, with this level of impact extending to relationships with their other children for just over half (57.6%). Carer relationship with the AI were also significantly or very significantly impacted for a majority (78.8%). Carers commonly (65.2%) reported significant or very significant impact on their work life. In terms of a global measure of quality of life, 89% reported significant or very significant impacts.

### Economic impacts of the ED on carers

Table 2 summarises the financial and other economic impacts reported by carers.

**Table 2:** Financial and other economic impacts reported by COSTS study carer participants (n = 121 unless otherwise stated)

Impact	n (%) or median (range)
Loss of income (past year/last year of ED, 2020 $NZ)^a^ (n = 87)	$11,650 ($200–$141,950)
Reduction in income (%)	13.6% (1.8–100%)
*Work/study hours (past/last year) (n* = *112)*	
None, no impact	29 (25.9%)
Reduced hours per week	5 h (0–40 h)
*Reduced productivity in work or study*	98 (90.7%)
Extent of reduced productivity (n = 90)	66% (0–86%)
Prevented engaging in regular work/study	23 (20.5%)
Need to take extended sick leave (at least 4 weeks) (n = 102)	38 (37.3%)
Publicly funded treatment—for those reporting additional out of pocket costs (n = 23)	$1000 ($150–$5000)
Private treatment costs (n = 55)^b^	$4000 ($150–$30,000)
Travel/accommodation/relocation costs within NZ (n = 47)	$750 (range $125–$300,000)
Had medical insurance to cover treatment costs (n = 116)	8 (6.6%)
Accessed finance/sold assets to pay for treatment (n = 103)	14 (12.6%)
Increased food costs related to binge eating (n = 28)	$1500 ($125–$10,400)
Medication costs (out of pocket) (n = 109)	$125 (0–$2250)

^a^Loss of income (past/last year) refers to the past year if current eating disorder (ED), or the final year of the ED if the person is recovered. All expenditure was converted to the equivalent in 2020 New Zealand dollars (NZ$)

^b^Some costs reported here (e.g., General Practitioner assessment treatment) may have been partially subsidised by the government

#### Lost income and productivity

Loss of income was reported for the past year if the ED was still current, or the final year of the ED if the AI was recovered. All expenditure was converted to the equivalent in 2020 New Zealand dollars ($NZ). Carers who answered these questions (n = 87) reported a median 13.6% (range 1.8–100%) reduction in income in that year, ranging from $200 to $141,950. Over 90% of participants reported impaired productivity, with the median percent reduction of 66%, but ranging from 0 to 86%. The median hours-lost per week was 5 h, but ranged from 0 to 40 h. Thirty-seven percent had to take extended leave (of more than 4 weeks) and 20.5% reported that they were unable to undertake work or study due to their AI’s ED.

#### Direct costs of private treatment

Forty-five percent of the participants (n = 55) reported paying for private treatment for their AI. The median cost was $4000 but was as high as $30,000. After general practitioners (55.4%), other disciplines seen were psychologists (45.5%), psychiatrists (44.6%), dieticians (43.8%) and counsellors (31.4%). The category ‘ED specialist’ was endorsed by 53.7% however as this was not specifically defined, the disciplines seen for treatment are unable to be determined but may have included other disciplines not listed such as nurses with eating disorder experience.

#### Travel, accommodation and relocation costs

Over half of the carers (n = 74, 59%) either did not answer this question or reported no costs for this item. As such, the costs reported will be an underestimate of true costs, as carers have obviously not considered transport to and from treatment or other travel expenses when almost all would have experienced these costs. Of those who did answer the question, there was marked variability in costs in this domain. Although the median cost was $750, the range was from $125 to $300,000. Only three participants reported costs over $100,000, and these all related to having to relocate their families closer to treatment services within the major cities.

#### Other out of pocket expenses

Almost all participants reported minimal medication expenditure (median was $125 per year, $0–$2250) for their affected individual. For the few participants reporting spending over $2000, all specified that this was primarily on vitamins and supplements for their affected individual.

Twenty-eight participants reported increased food costs related to binges. The median cost was $1500 (range $125—$10,400).

#### Financing treatment

Only 6.6% of the 109 respondents reported having health insurance that covered treatment. Of 103 responses, 12.4% (n = 14) reported that they had to access finance or sell assets to pay for treatment for their AI, with 11 (9.1% of the whole sample) of these carers needing to access over $10,000.

## Discussion

This study highlights the pervasive burden of care in New Zealand for caregivers of people with eating disorders. The typical carer experience is one of significant impacts on multiple relationships in their life including their working life. Not unexpectedly, significantly impaired family life is the norm. The effect on caregivers “wearing all the hats” (page 5, [48]) clearly ripples out to all family members who may feel overlooked or less cared for. EDs, especially AN, directly affect family dynamics including effects on siblings [49]. The continued and consistent findings about burden on family members supports the need for routine evaluation of family effects, while being careful not to blame family disfunction as causative.

The most frequently reported deleterious relationship experienced by the caregiver is with the AI themselves. Strained personal interactions between caregivers and the AI are well known to occur. While the current study did not explore the consequences of these and other reported impacts, other studies point to detrimental repercussions including maintaining the eating disorder [50]. A recent study however suggests that rates of positive expressed emotions and attitudes are just as frequent and that some forms of positive emotional “over”-involvement may be associated with a more favourable prognosis [51]. Though, even this may take its toll where, for example, carers do this at the expense of other family members who may feel neglected, fearful or even guilty about their role [52]. This points to the complexities of understanding how impact networks need to be understood and the limitation of considering impact components in isolation.

The study also provides a window into the broader impacts, in that finding or accessing treatment is a significant problem for most. Treatment inaccessibility is not unique to New Zealand. Studies in comparable countries such as Australia have found likewise [53]. However, delayed access to treatment may be one of many factors contributing to illness duration and outcomes [54] and accumulating carer impacts, leaving families having to compensate by becoming default professional caregivers. Even if families reach out to private treatment, in this study only a few had access to health insurance which would cover aspects of this. Further studies documenting the economic impacts on caregivers is needed if resources to offset these costs are to be successfully argued for in EDs, as they have in other severe and enduring health conditions. While New Zealand organisational models of service delivery have been articulated [55] little has changed in terms of access to services in the intervening decade.

Inevitably the reported psychosocial and economic impacts could be expected to intertwine. For example, for those with especially high income impacts due to needing to reduce or cease work, this may contribute to relationship strains (subjective burden) and, in turn, mental wellbeing. In turn, this may detrimentally affect parenting of other siblings and opportunities lost by those siblings. As another example, disrupting careers through taking extended leave not only has obvious economic impacts, but may have other repercussions such as reduced access to job satisfaction and other relational aspects of quality of work life. However, the relationship between subjective and objective burden is likely to be complex, including gender variables likely to influence this [8]. This highlights the importance of going beyond a descriptive study in order to explore the nuances, and thereafter tailoring support.

## Limitations

There were a number of limitations of this research, foremost of which were measurement and sampling biases. Firstly, the carer impacts were self-reported: this may have either under-estimated the actual impact (due to becoming habituated to the carer role over time or not recalling detailed costs of long illness episodes) or overestimated it (due to the immediate distress with carer demands at the time of the study). However, subjective self-report is a risk in many burden studies, including in quality of life research more generally. Countering this, emotional impact is a subjective concept so self-report is a valid means to assess this. The self-reporting limitation also affected reporting on recovery status. Caregivers may have quite different definitions to patients [56] and researchers [57], thereby significantly affecting the rates reported. On a similar note, none of the ED diagnoses reported by the caregivers were matched with clinical diagnoses. This is a common issue when recruiting caregivers through support groups and networks rather than through clinics.

A small number of participants (n = 3) were recruited during the COVID-19 pandemic disruptions/lockdown in New Zealand, including when there were disrupted access to health services and supports. This may have increased perceived carer burden [58], particularly in regard to the psychological measures. However, given the relative duration of EDs, for these participants this will have only skewed a small proportion of carer-giving in the overall time as a care-giver. Finally, the small sample prevented any consideration of ethnicity. New Zealand Māori are known to experience health inequities [59, 60] and are less likely to receive treatment [61] and thus likely to extend to greater carer impacts.

Unfortunately, data regarding some financial questions (e.g., travel, relocation, accommodation) were limited because a majority of participants elected not to provide an answer. This could have arisen because the question was perceived as irrelevant, especially in a small country where major geographical relocation to access mental health services is less likely than some other countries. Obtaining more comprehensive information may require alternative methodologies such as interviews.

Various sampling biases may have skewed results. Firstly, there may have been over-recruitment of those who felt especially burdened and felt motivated to take part because the topic resonated with them. Further, the online format may have favoured younger and the more online agile carers.

The analysis is also largely limited to those caring for people with AN, of whom many still had an ongoing ED. Theoretically at least, the impacts for these carers is still accumulating, rendering current data as a likely underestimate. The recruitment of mainly carers of people with anorexia nervosa is also a sampling bias found in other studies (e.g., [62] suggesting also a need to use other recruitment strategies if the impacts of care in other EDs is the focus of study. Related to this sampling bias, male carers were largely absent amongst participants. Assessing the impact on fathers is under-researched in other studies as well. While mothers tend to take greater responsibility to caring for an AI, and thus are understandably more responsive to carer impacts studies, other factors may also be preventing father engagement including factors which also may explain lower involvement in treatment in any case.

The sampling bias of predominantly recruiting female carers is not unique to this study in that female participants tend to outnumber male in eating disorder research on carer impact and carer interventions [63, 64]. Future research should consider designs which can recruit more male caregivers, thereby enabling better gender comparisons, as has occurred in other areas of carer impact research [10]. Understanding the impacts on fathers is important given that they are already less likely to use social support strategies [65]. Finally, the study collected limited information on costs: more comprehensive information may require an alternative methodology such as interviews.

While acknowledging these limitations, to the best of our knowledge, this is the first quantitative study exploring the range of significant impacts experienced by carers of those with EDs in New Zealand. The study provides important information about difficulties with treatment barriers, financial and psychosocial impacts borne by this group of carers. These findings point to issues for funders and services providers to consider regarding unmet needs in service delivery and support that have a substantial impact on those affected and their families. Greater support is needed for carers who play a pivotal role during treatment and in continuing to support recovery for their family member affected by the ED.

## Future directions

The current study did not explore potentially positive impacts on carers. However, research describing the possibility of positives (e.g. family members ‘pulling together’ [66]) and silver linings [18] suggest that more studies could seek to explore how these impacts interweave with the negative. For example, how might these positive impacts differ from the types of initial positive responses reported in other traumatic contexts, where people report pulling together, in the short term at least. Capturing the true extent of carer impacts will require more complex research designs conducted over time, thereby refining the peak events or illness stages which especially impact on these impacts. In turn this can help target the timing and type of interventions for carers. Finally, at present there is little known about the impact on carers of those with less common eating disorders such as Avoidant/Restrictive Food Intake Disorder (AFRID), leaving scope for research in this area.

## Conclusions

In summary, this study of carers in New Zealand found longstanding and extensive impacts, extending out to those around them. Most of those they care for get access to public treatment at some stage, but the financial and economic impacts on carers are likely to take years to recoup. Better interventions and supports for carers are much needed in New Zealand. Further, studies utilising more comprehensive research methodology are needed to provide more detailed and accurate accounts of the impacts, as well as any positive repercussions, over the long course of the caregiving role.

## Data Availability

The data that support the findings of this study are available on request from Associate Professor Jennifer Jordan (jenny.jordan@otago.ac.nz).

## Abbreviations

AN: Anorexia nervosa
AFRID: Avoidant/restrictive food intake disorder
AI: Affected individual
BN: Bulimia nervosa
BED: Binge eating disorder
ED: Eating disorder
EDSIS: Eating Disorders Symptom Impact Scale
EDANZ: Eating Disorders Association of New Zealand NZ
IEQ-EU: Involvement Evaluation Questionnaire-European Version New Zealand
OSFED: Other specified feeding or eating disorder
USD: United Stated dollars
UK: United Kingdom
